# Asthma and Memory Function in Children

**DOI:** 10.1001/jamanetworkopen.2024.42803

**Published:** 2024-11-11

**Authors:** Nicholas J. Christopher-Hayes, Sarah C. Haynes, Nicholas J. Kenyon, Vidya D. Merchant, Julie B. Schweitzer, Simona Ghetti

**Affiliations:** 1 Center for Mind and Brain, University of California, Davis; 2 Department of Psychology, University of California, Davis; 3 Department of Pediatrics, University of California Davis School of Medicine, Sacramento; 4 Department of Internal Medicine, University of California Davis School of Medicine, Sacramento; 5 Department of Psychiatry and Behavioral Sciences, University of California Davis School of Medicine, Sacramento; 6 MIND Institute, University of California Davis School of Medicine, Sacramento

## Abstract

**Importance:**

Asthma is a chronic respiratory disease affecting approximately 5 million children in the US. Rodent models of asthma indicate memory deficits, but little is known about whether asthma alters children’s memory development.

**Objective:**

To assess whether childhood asthma is associated with lower memory abilities in children.

**Design, Setting, and Participants:**

This cohort study used observational data from the Adolescent Brain Cognitive Development (ABCD) Study, a multisite longitudinal investigation that began enrollment in 2015. Approximately 11 800 children aged 9 to 10 years were enrolled at baseline with follow-up at 1 and 2 years. Participants were selected based on exposures described subsequently to determine longitudinal and cross-sectional associations between asthma and memory. Data were analyzed from Month year to Month year.

**Exposures:**

Asthma was determined from parent reports. For the longitudinal analysis, children were selected if they had asthma at baseline and at the 2-year follow-up (earlier childhood onset), at the 2-year follow-up only (later childhood onset), or no history of asthma. For the cross-sectional analysis, children were selected if they had asthma at any time point, or no history of asthma. The comparison group of children with asthma history was matched on demographic and health covariates for each analysis.

**Main Outcomes and Measures:**

The primary outcome was episodic memory. Secondary outcomes included processing speed, inhibition and attention.

**Results:**

Four hundred seventy-four children were included in the longitudinal analysis (earlier childhood onset: 135 children; mean [SD] age, 9.90 [0.63] years; 76 [56%] male; 53 [28%] Black, 29 [21%] Hispanic or Latino, and 91 [48%] White; later childhood onset: 102 children; mean [SD] age 9.88 [0.59] years; 54 [53%] female; 22 [17%] Black, 19 [19%] Hispanic or Latino, and 83 [63%] White; comparison: 237 children; mean [SD] age, 9.89 [0.59] years; 121 [51%] male; 47 [15%] Black, 48 [20%] Hispanic or Latino, and 194 [62%] White). Children with earlier onset of asthma exhibited lower rates of longitudinal memory improvements relative to the comparison group (β = −0.17; 95% CI, −0.28 to −0.05; *P* = .01). Two thousand sixty-two children were selected for the cross-sectional analysis (with asthma: 1031 children; mean [SD] age, 11.99 [0.66] years; 588 [57%] male; 360 [27%] Black, 186 [18%] Hispanic or Latino, and 719 [54%] White; without asthma: 1031 children; mean [SD] age 12.00 [0.66] years; 477 [54%] female; 273 [21%] Black, 242 [23%] Hispanic or Latino, and 782 [59%] White). Children with asthma (1031 children) showed lower scores on episodic memory (β = −0.09; 95% CI, −0.18 to −0.01; *P* = .04), processing speed (β = −0.13; 95% CI, −0.22 to −0.03; *P* = .01), and inhibition and attention (β = −0.11; 95% CI, −0.21 to −0.02; *P* = .02).

**Conclusions and Relevance:**

In this cohort study, asthma was associated with memory difficulties in children, which may be more severe if asthma onset is earlier in childhood and may extend to executive function abilities.

## Key Points

**Question:**

Is childhood asthma associated with lower memory and cognitive function in children?

**Findings:**

This cohort study of 474 children found that children with earlier asthma onset showed slower development of episodic memory. More generally, children with any asthma exposure showed lower scores on measures of episodic memory and executive function.

**Meaning:**

These findings suggest that asthma is associated with memory and executive function difficulties in children.

## Teaser

This cohort study evaluates the association between childhood asthma and memory function among children aged 9 to 10 years.

## Introduction

Asthma is one of the most common chronic diseases in childhood, affecting approximately 6.5% of children in the US, with a higher frequency among males.^1,2,3^ Symptoms of asthma are largely respiratory, and may include wheezing, coughing, and shortness of breath.^4,5^ Recent studies from rodent models suggested that asthma also results in neural injury and associated memory declines.^6,7,8,9,10^ Chronic asthma frequently emerges in childhood, but whether asthma is associated with memory difficulties in children is largely unknown.

Although the exact pathophysiology of asthma is unclear, it is defined by an ongoing inflammatory process that is not limited to the lungs, and may extend to the brain as neuroinflammation.^10,11,12,13^ Rodent models indicate that asthma results in neural injury in the hippocampus, a brain structure with high vulnerability to neuroinflammatory responses.^8,14,15,16,17^ Moreover, respiratory difficulties may result in cerebral hypoxia episodes, which may lead to hippocampal injury.^8,18,19,20^ Finally, rodent models of asthma have revealed memory deficits in tasks requiring hippocampal integrity.^8,19,20^

Asthma has a peak incidence between the ages of 4 and 12 years.^1,2,3^ To date, little is known about memory functioning in children with asthma. Previous studies^21^ demonstrated that children with asthma who are treated with higher doses of corticosteroids exhibited reduced verbal memory compared with children receiving lower doses, but the absence of a comparison group of children without asthma precludes any conclusions about associations between asthma and memory abilities. Other studies^18,22,23^ also reported difficulties in attention, executive function, and visual and working memory, but these studies did not account for confounding effects of socioeconomic factors that might affect both the probability of developing asthma and cognitive difficulties. It is critical to understand whether asthma is associated with memory or other cognitive difficulties during childhood while accounting for potentially confounding variables, given the importance of cognitive abilities for academic achievement and occupation prospects.^24,25,26,27^

Research concerning other medical conditions (eg, type 1 diabetes) has shown that children who experience complications (eg, diabetic ketoacidosis) at a younger age may be particularly vulnerable to cognitive difficulties.^28,29,30^ However, disease duration may also be critical, given that in adults, asthma duration is associated with changes in brain structure and function.^31,32,33,34^ Therefore, we hypothesize that children who experience asthma at an earlier age may be at greater risk for memory difficulties.

We leveraged longitudinal data collected from the Adolescent Brain and Cognitive Development (ABCD) Study to assess whether children with asthma exhibit lower memory performance. Episodic memory, the ability to remember past events with specific detail, requires hippocampal integrity, as do memory processes targeted in rodent models.^8,19,20^ This ability improves substantially during childhood.^35^ Thus, children with earlier asthma onset are hypothesized to show slower developmental improvement of memory over time compared with children with later onset asthma and in contrast to a matched comparison group of children without asthma. Based on findings in adult humans,^9,31^ we also hypothesized that asthma would be associated with reduced performance in tasks assessing executive function and processing speed. Thus, children who have had asthma at any point are hypothesized to show lower episodic memory and executive function performance, regardless of timing and duration, relative to a comparison group of children without asthma history.

## Methods

### Sample

The present study was reported using Strengthening the Reporting of Observational Studies in Epidemiology (STROBE) reporting guidelines.^36^ We selected our study sample from the ABCD Study, a nationwide National Institutes of Health (NIH)–funded longitudinal study of brain development and child health from 21 sites in the US (approximately 11 800 children aged 9-10 years enrolled at baseline).^37^ Procedures of the ABCD Study were approved under the institutional review board of the University of California, San Diego. Parents or legal guardians provided written informed consent before participation; children provided written assent. Race and ethnicity demographics (American Indian, Asian, Black, Hispanic or Latino, White, or Other race [Alaska Native, Guamanian, Native Hawaiian, Samoan, and Other Pacific Islander]) are reported using guidelines from the NIH and were determined from parent or guardian report. Race and ethnicity were described to assess the variability in the distribution of racial and ethnic groups across asthma status in the ABCD Study. For the current report, we focused on data collected at the baseline, 1-year follow-up, and 2-year follow-up time points (see Garavan et al^37^ for further information on design and procedures).

### Sampling Criteria

Inclusion criteria for children with asthma was based on parent reports of asthma attacks or asthma-related medical treatment. These asthma indicators were combined in 2 ways to generate 2 separate samples: 1 longitudinal sample and 1 cross-sectional sample. For the longitudinal sample, we focused on time points for which indicators of both asthma and cognitive performance were available, and we distinguished between children whose parents reported asthma indicators at the baseline and at the 2-year follow-up time point (earlier childhood onset group), and those who reported asthma indicators only at the 2-year follow-up time point (later childhood onset group). For the cross-sectional sample, we identified the asthma group as those children whose parents reported indicators of asthma at any time point (baseline, 1-year follow-up, or 2-year follow-up).

The comparison group included participants with no reports of asthma and was selected separately for the longitudinal and cross-sectional samples. Exclusion criteria included any report of schizophrenia, autism spectrum disorder, cerebral palsy, intellectual disability, tumor, aneurysm, hemorrhage, hematoma, seizure or epilepsy, multiple sclerosis, head or brain injury, diabetes, lead poisoning, heart conditions, and alcohol or substance use disorder.

### Asthma Status Indicators and Cognitive Assessments

Indicators of asthma were derived from parent report of their child’s experienced asthma attacks (continuous variable ranging from 0 to 40) and medical treatment for asthma (categorical variable of yes or no). The use of these indicators was previously validated, showing they are highly associated with polygenic risk score of asthma.^38^ An examination of reported medications confirms higher frequency of use of asthma-related medications (ie, bronchodilators, corticosteroids, or other anti-inflammatories) among children with asthma vs the comparison group in both the longitudinal (eFigure 1 and eTables 1-2 in Supplement 1) and cross-sectional samples (eFigure 2 and eTables 3-4 in Supplement 1). We selected a set of cognitive measures administered in the ABCD Study from the NIH Toolbox^39^ based on hypothesized associations with asthma.^8,9,19,20,31^

#### Picture Sequence Memory Test

The picture sequence memory test was the primary outcome measure because it assesses hippocampus-dependent episodic memory, necessitating retention of arbitrary sequences of objects and activities.^39,40,41^ Standardized scores reflect the cumulative number of adjacent picture sets remembered correctly across the 15-picture sequences.

#### Pattern Comparison Processing Speed Test

This measure assesses processing speed with pairs of object pictures where participants are asked to make a same or different judgment.^39,42^ Standardized scores reflect the timed number of correct responses.

#### Flanker Inhibitory Control and Attention Test

This is an executive function measure of response inhibition and attention. Participants view arrays of arrows (5) pointing to either the left or the right. Participants sought to select the correct direction of the central arrow (the target), under conditions in which the center arrow points in the same or opposite direction as the 4 other flanking arrows. Standardized scores are computed based on both accuracy and response time.^39,43^

### Statistical Analysis

#### Participant Matching

We used Matchit version 4.5.3 implemented in R version 4.4.1 (R Project for Statistical Computing)^44,45^ with a 1:1 nearest neighbor propensity score matching procedure (further details in eMethods in Supplement 1)^46^ without replacement to select comparison groups for our analyses. The following covariates were included in this procedure: age, sex, combined parental income, and other health indicators, including allergies and bronchitis due to their high frequency in asthma. For longitudinal analyses, asthma subgroups (ie, earlier childhood onset and later childhood onset) were collapsed into 1 asthma group for the purpose of selecting a comparison group from all candidate children without any asthma indicators in the ABCD Study. We did not match for race or ethnicity in order to capture variability in the distribution of racial and ethnic groups across asthma status in the ABCD Study; this approach helps attenuate selection biases.^47^ Moreover, race and ethnicity are typically associated with socioeconomic variables such as income, which is accounted for in our analyses, thus countering the use of race and ethnic groups as proxies for socioeconomic factors.^47^ Variance related to socioeconomics was assessed by the combined parental income, which is a resource-based proxy measure of socioeconomic status. Hypothesis testing of count variables were conducted using the Pearson χ^2^ test, and tests without specific hypotheses used omnibus analysis of variance.

#### Longitudinal Models

Longitudinal analyses were conducted using linear growth models in R (packages lme4 versions 1.1-35.1 and lmertest versions 3.1-3*).* We examined episodic memory as a function of group membership (earlier childhood onset, later childhood onset, and comparison group), age at baseline, change in age (from baseline to 2-year follow-up), and their interaction. All interaction effects between change in age and participant group specify the comparison group as the reference group. In addition, the remaining covariates used to match the groups (ie, sex, combined parental income, and other health indicators) were included to ensure the model accounted for any association of these covariates with episodic memory beyond group membership.^48^ All growth models included a random intercept for participant. Age at the baseline time point was centered at the minimum age of the sample (9 years). Analogous models were conducted for the secondary analyses with processing speed and inhibition and attention. Additionally, to determine if propensity score matching procedures introduced bias by excluding participants,^49^ we re-estimated all longitudinal models but with the entirety of the comparison group candidates.

#### Cross Sectional Models

Cross-sectional analyses were carried out using linear regression models in R (packages lme4 versions 1.1-35.1 and lmertest versions 3.1-3). We assessed differences in episodic memory as a function of group membership (asthma vs reference comparison group). The remaining covariates used to match the groups (ie, sex, combined parental income, and other health indicators) were also included. Cross-sectional models included nested random intercepts for family under research site.

#### Model Assessment

All models were inspected for linearity, homoscedasticity, and normality with plots of the residuals against fitted values. Normalized cognitive scores were used in all analyses. To determine whether models were overfitted by including all covariates used for matching, we applied the R step function (stats version 4.3.3). Effect estimates were deemed significant at *P* < .05.

## Results

### Longitudinal Analyses

We identified 237 children with asthma based on inclusion and exclusion criteria (earlier childhood onset: 135 children; mean [SD] age, 9.90 [0.63] years; 76 [56%] male; 15 [8%] American Indian, 12 [6%] Asian, 53 [28%] Black, 29 [21%] Hispanic or Latino, and 91 [48%] White; later childhood onset: 102 children; mean [SD] age 9.88 [0.59] years; 54 [53%] female; 5 [4%] American Indian, 10 [8%] Asian, 22 [17%] Black, 19 [19%] Hispanic or Latino, and 83 [63%] White). We then derived a comparison group of children without asthma (237 children; mean [SD] age, 9.90 [0.60] years; 121 [51%] male; 22 [7%] American Indian, 26 [8%] Asian, 47 [15%] Black, 48 [20%] Hispanic or Latino, and 194 [62%] White) who were successfully matched to the asthma group on all covariates at the baseline time point (eFigures 3-4 and eTable 5 in Supplement 1). The groups were statistically similar in pubertal status (eTable 6 in Supplement 1). Demographic information and descriptive statistics for cognitive data are provided in Table 1. Asthma group membership was not evenly distributed across racial groups (χ^2^_8_ = 19.05; *P* = .02), but was evenly distributed across ethnic groups (χ^2^_2_ = 0.29; *P* = .86). Parental income was associated with racial group (eTable 7 in Supplement 1) and was accounted for in our longitudinal models.

**Table 1.** Longitudinal Sample (Baseline and 2-Year Follow-Up) Baseline Demographics and Cognitive Measures

Demographics	Participants, No. (%)		
	Earlier onset (n = 135)	Later onset (n = 102)	Comparison (n = 237)
Age, mean (SD), y	9.90 (0.63)	9.88 (0.59)	9.90 (0.60)
Income scale, mean (SD)^a^	6.38 (2.61)	7.66 (1.99)	7.15 (2.28)
Sex			
Female	59 (44)	54 (53)	116 (49)
Male	76 (56)	48 (47)	121 (51)
Race and ethnicity^b^			
American Indian	15 (8)	5 (4)	22 (7)
Asian^c^	12 (6)	10 (8)	26 (8)
Black	53 (28)	22 (17)	47 (15)
Hispanic or Latino	29 (21)	19 (19)	48 (20)
White	91 (48)	83 (63)	194 (62)
Other race^d^	18 (9)	12 (9)	23 (7)
Other health^b^	98 (73)	67 (66)	165 (69)
Cognitive measures, mean (SD)			
Episodic memory^e^	103.47 (11.79)	102.19 (12.08)	103.23 (10.20)
Processing speed^f^	87.76 (14.53)	88.98 (13.76)	89.22 (14.31)
Inhibition and attention^g^	92.94 (9.74)	95.62 (9.90)	94.69 (8.17)

^a^ Income scale: 1 = less than $5000; 2 = $5000 through $11 999; 3 = $12 000 through $15 999; 4 = $16 000 through $24 999; 5 = $25 000 through $34 999; 6 = $35 000 through $49 999; 7 = $50 000 through $74 999; 8 = $75 000 through $99 999; 9 = $100 000 through $199 999; and 10 = $200 000 and above.

^b^ Counts for race and ethnicity and other health may not sum to group totals due to incomplete or multirace reporting.

^c^ Asian includes Asian Indian, Chinese, Filipino, Japanese, Korean, Vietnamese, and Other Asian.

^d^ Other race includes Alaska Native, Guamanian, Native Hawaiian, Samoan, and Other Pacific Islander.

^e^ Episodic memory range, 79 to 136.

^f^ Processing speed range, 49 to 136.

^g^ Inhibition and attention range, 55 to 114.

**Figure 1.**
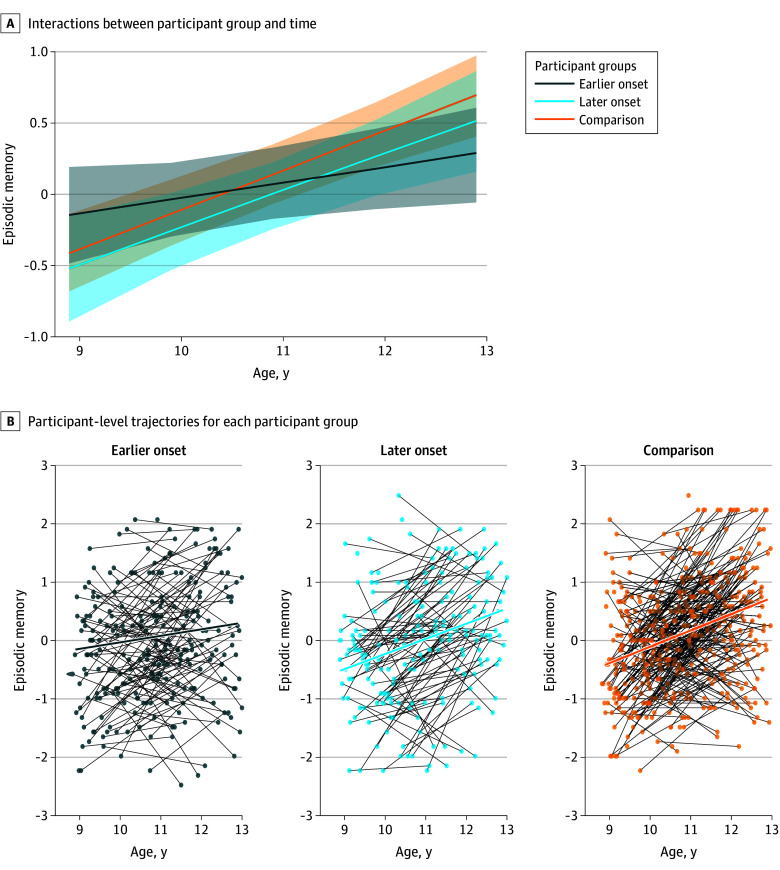
Developmental Trajectories of Memory Abilities as a Function of Participant Group A, Interactions between participant group and time with lines-of-best-fit for fixed effects and a binary underlay of individual bootstrapped interval estimates (1000 simulations) around estimate lines, and B, participant-level trajectories for each participant group from which estimates shown in panel A are obtained.

Episodic memory improved over time overall (change in age: β = 0.28; 95% CI, 0.21 to 0.35; *P* < .001), but the earlier childhood onset group exhibited lower rates of improvements in episodic memory abilities relative to the comparison group (change in age × earlier onset: β = −0.17; 95% CI, −0.28 to −0.05; *P* = .01) (Figure 1; eTable 8 in Supplement 1), but no such difference was observed between the later childhood onset group and the comparison group (change in age × later onset: β = −0.02; 95% CI, −0.15 to 0.10; *P* = .75).

We then tested longitudinal models for processing speed and inhibition and attention. Similar to episodic memory abilities, we found improvements over time in both processing speed (change in age: β = 0.39; 95% CI, 0.32-0.45; *P* < .001) and inhibition and attention (change in age: β = 0.29; 95% CI, 0.21-0.36; *P* < .001), but no significant group differences were found (eFigure 5 and eTable 8 in Supplement 1).

These results were unaffected by the inclusion and exclusion of the outlined covariates (eTable 9 in Supplement 1) and use of alternative asthma indicators (ie, asthma attacks) (eTable 10 and eFigure 6 in Supplement 1). The results were replicated with models using the entirety of the ABCD sample comparison group candidates (ie, children without any asthma indicators at any visit; 8414 in sample at baseline) without implementing any matching procedure (eTable 11 in Supplement 1).^49^

### Cross-Sectional Analyses

We identified children with a history of asthma (1031 children; mean [SD] age, 11.99 [0.66] years; 588 male [57%]; 62 [5%] American Indian, 81 [6%] Asian, 360 [27%] Black, 186 [18%] Hispanic or Latino, and 719 [54%] White) based on inclusion and exclusion criteria described earlier, and a comparison group of children without asthma (1031 children; mean [SD] age 12.00 [0.66] years; 477 [54%] female; 63 [5%] American Indian, 69 [5%] Asian, 273 [21%] Black, 242 [23%] Hispanic or Latino, and 782 [59%] White) who were successfully matched on all covariates at the 2-year follow-up time point (eFigures 3 and 7 and eTable 5 in Supplement 1). The groups were statistically similar in pubertal status (eTable 12 in Supplement 1). Demographic information for this sample as well as descriptive statistics for cognitive data are provided in Table 2. Asthma group membership was not evenly distributed across racial groups (χ^2^_4_ = 17.75; *P* = .001) nor across ethnic groups (χ^2^_1_ = 8.92; *P* = .003). Parental income was associated with racial group (eTable 13 in Supplement 1) and was accounted for in the cross-sectional models.

**Table 2.** Cross-Sectional Sample (2-Year Follow-Up) Demographics and Cognitive Measures

Demographics	Participants, No. (%)	
	Asthma (n = 1031)	Comparison (n = 1031)
Age, mean (SD), y	11.99 (0.66)	12.00 (0.66)
Income scale, mean (SD)^a^	6.74 (2.59)	6.66 (2.58)
Sex		
Female	443 (43)	477 (54)
Male	588 (57)	554 (46)
Race and ethnicity^b^		
American Indian	62 (5)	63 (5)
Asian^c^	81 (6)	69 (5)
Black	360 (27)	273 (21)
Hispanic or Latino	186 (18)	242 (23)
White	719 (54)	782 (59)
Other race^d^	107 (8)	130 (10)
Other health (allergies)^b^	557 (54)	557 (54)
Cognitive measures		
Episodic memory^e^	107.40 (12.79)	108.64 (12.99)
Processing speed^f^	101.21 (15.52)	103.50 (15.63)
Inhibition and attention^g^	99.18 (8.01)	100.07 (7.83)

^a^ Income scale: 1 = less than $5000; 2 = $5000 through $11 999; 3 = $12 000 through $15 999; 4 = $16 000 through $24 999; 5 = $25 000 through $34 999; 6 = $35 000 through $49 999; 7 = $50 000 through $74 999; 8 = $75 000 through $99 999; 9 = $100 000 through $199 999; and 10 = $200 000 and above.

^b^ Counts for race and ethnicity and other health may not sum to group totals due to incomplete or multirace reporting.

^c^ Asian includes Asian Indian, Chinese, Filipino, Japanese, Korean, Vietnamese, and Other Asian.

^d^ Other race includes Alaska Native, Guamanian, Native Hawaiian, Samoan, and Other Pacific Islander.

^e^ Episodic memory range, 76 to 136.

^f^ Processing speed range, 47 to 155.

^g^ Inhibition and attention range, 76 to 133.

**Figure 2.**
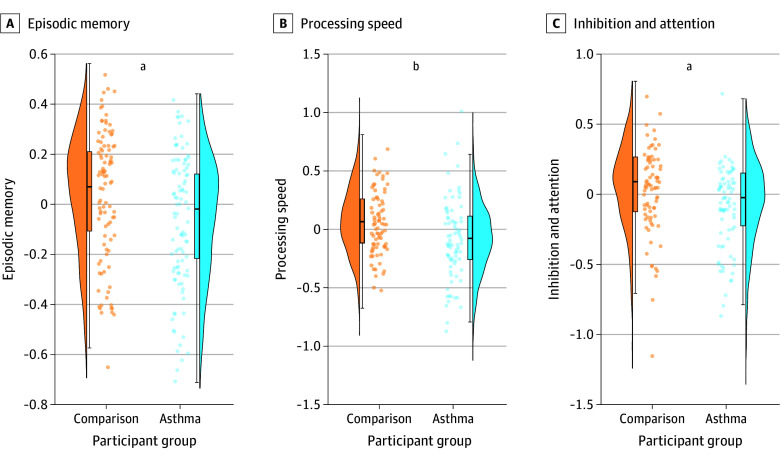
Episodic Memory, Processing Speed, and Inhibition and Attention in Children With and Without a History of Asthma Each raincloud plot from left to right shows the participant-level data for each cognitive measure as a function of participant group. Each plot includes density and cumulative probability values for the quantiles with the median (box plot: 25%, 50%, and 75%), and upper and lower limits (whiskers: 2, 98%). To reduce visual burden, 10% of individual participant-level data points were randomly sampled and plotted. In raincloud plots, participant group is denoted on the x-axis, and scaled estimate scores for each cognitive measure (episodic memory, processing speed, inhibition and attention) are shown on the y-axis. ^a^*P* < .05. ^b^*P* < .01.

We found that children with an asthma history performed worse on episodic memory (β = −0.09; 95% CI, −0.18 to −0.01; *P* = .04), processing speed (β = −0.13; 95% CI, −0.22 to −0.03; *P* = .01), and inhibition and attention measures (β = −0.11; 95% CI, −0.21 to −0.02; *P* = .02) (Figure 2; eTable 14 in Supplement 1) relative to the comparison group of children with no asthma history. These results were unaffected by the inclusion and exclusion of the outlined covariates using model selection methods (eTable 15 in Supplement 1).

## Discussion

The results from this cohort study provide initial evidence that children who experience asthma are more likely to perform worse on memory tasks. In a longitudinal analysis, children with earlier onset of asthma exhibited reduced developmental improvement in episodic memory over time relative to the comparison group. Developmental improvement in children with later onset of asthma did not differ from the comparison group. We did not find any group differences in developmental trajectories of processing speed or inhibition and attention.

Asthma occurring at a younger age, as is the case for the earlier childhood onset group, may be particularly likely to perturb neurodevelopment,^30^ especially in brain regions that demonstrate ongoing plasticity and vulnerability to environmental input (eg, the hippocampus).^16^ Moreover, children with earlier onset of asthma in this study experienced asthma for a longer period of time. Asthma is a disease of airways and systemic inflammation, including neuroinflammation.^10,11,12,13^ Prolonged inflammation due to longer duration may result in detrimental neuroinflammatory responses, which may in turn disrupt neural processing and manifest in the form of cognitive dysfunction, consistent with results from rodent models of asthma.^8,19,20^ Future studies including additional assessment points will help disentangle effects of early exposure (ie, age of onset) from effects associated with the duration of exposure.

We replicated results from the longitudinal approach using a cross-sectional analysis with a markedly larger sample. Here, we found that children with any asthma history not only showed lower scores for episodic memory, but also for processing speed and inhibition and attention. Though rodent models of asthma have not assessed other behaviors beyond learning and memory,^8,19,20^ studies in human adults have reported associations between disease-related factors and a broader array of functions including attention, executive function, processing speed, and visual-spatial processing.^9,31^ It is possible that associations between asthma and developmental trajectories emerge earlier for memory, perhaps due to its sensitivity to subtle hippocampal injury.^16^ Future studies including additional time points of data will also be able to address this open question. Future analyses should also examine whether the extent of asthma symptoms over time increases risk of ADHD given our cross-sectional evidence of associations between asthma and cognitive functions typically affected in ADHD (ie, inhibition and attention).^50^

### Limitations

There are several limitations in this study. The present study did not directly rely on medical records. Thus, we were unable to precisely assess the severity of the disease or its exact onset and duration. However, asthma indicators in the ABCD Study have been validated in other research.^38^ Moreover, this study did not examine whether or how prescription corticosteroids affect neurocognitive development or their interactions with asthma, despite their frequent use for asthma treatment and evidence of their adverse effects on cognition.^19,51,52^ Use of medication and dosage were not probed consistently over time precluding the examination of associations between extent of exposure to medications and cognitive functioning. Future studies should seek comprehensive strategies to examine dosage and frequency of use, since these variables seem critical based on experimental findings in nonhuman research. The ABCD Study was designed as a national study of neurocognitive development, and therefore was not optimized to examine associations between asthma and cognition. As such, these associations ought to be evaluated in the context of large variability on variables that may be associated with both asthma and cognitive measures. For example, future research may recruit prospectively for key factors including asthma severity, medication regimen, and exposure to environmental pollutants. Additionally, asthma group membership was not independent of racial and ethnic groups. We used combined parental income to avoid the use of race and ethnicity as proxies for socioeconomic variables,^47^ but future studies should examine additional factors that may be associated with race and ethnicity and contribute to effects of asthma on neurocognitive development (eg, pollution and access to medical care). Determination of asthma should be expanded to include not only parent report, but also clinical measures such as lung function tests and asthma-specific questionnaires on symptoms and disease management. Lastly, practice effects exist in some cognitive measures used here,^53^ which might alter trajectory estimates. However, participant groups were similar in this cohort study schedule and repeated experience of all tasks, and age at baseline and 2-year-follow-up, reducing the potential that practice explained reported group differences. The number of available assessments should be considered in future analyses as future loss to follow-up may differ based on asthma group.

## Conclusions

Despite these limitations, the current study provides novel evidence that children with asthma exhibit altered memory functioning during development, in addition to consequences extending to other cognitive domains. These results accentuate the need to examine this population more closely to understand the full extent to which asthma influences neurodevelopment.

## Notes

Supplement 1.
eMethods.
**eTable 1. **Longitudinal Sample (Baseline and 2-Year Follow-Up) Most Frequent Medications at Baseline **eTable 2.** Longitudinal Sample (Baseline and 2-Year Follow-Up) Medication Classes for Asthma Disease Management at Baseline **eTable 3.** Cross-Sectional Sample (2-year Follow-Up) Most Frequent Medications **eTable 4.** Cross-Sectional Sample (2-Year Follow-Up) Medication Classes for Asthma Disease Management **eTable 5.** Sample Derivation With Covariate Matching **eTable 6.** Longitudinal Sample Age and Pubertal Status at Baseline and 2-Year Follow-Up **eTable 7.** Longitudinal Analyses of Combined Parental Income Regressed on Race **eTable 8.** Longitudinal Analyses of Asthma Effects on Cognitive Outcomes Using a Matched Comparison Group **eTable 9.** Longitudinal Analyses of Asthma Effects on Cognitive Outcomes Using a Matched Comparison Group and Data-Driven Model Selection **eTable 10.** Longitudinal Sample Sensitivity Analyses Examining Developmental Trajectories of Cognitive Outcomes as a Function of Asthma Attacks **eTable 11.** Longitudinal Analyses of Asthma Effects on Cognitive Outcomes Across the ABCD Sample **eTable 12.** Cross-Sectional Sample Pubertal Status at 2-Year Follow-up **eTable 13.** Cross-Sectional Analyses of Combined Parental Income Regressed on Race **eTable 14.** Cross-Sectional Analyses of Asthma Effects on Cognitive Outcomes Using a Matched Comparison Group **eTable 15.** Cross-Sectional Analyses of Asthma Effects on Cognitive Outcomes Using a Matched Comparison Group and Data-Driven Model Selection **eFigure 1.** Longitudinal Sample (Baseline and 2-Year Follow-Up) Most Frequent Medications at Baseline **eFigure 2.** Cross-Sectional Sample (2-Year Follow-Up) Most Frequent Medications **eFigure 3.** Covariate Balance for Derivation of Asthma Comparison Groups **eFigure 4.** Longitudinal Sampling Procedure **eFigure 5.** Developmental trajectories of Processing Speed, and Inhibition and Attention **eFigure 6. **Longitudinal Sample Sensitivity Analyses Examining Developmental Trajectories of Cognitive Outcomes as a Function of Asthma Attacks **eFigure 7.** Cross-Sectional Sampling Procedure
eReferences.

Supplement 2.
Data Sharing Statement
