# Supplementary material for: Asthma and Memory Function in Children
Source: JAMA Netw Open. 2024 Nov 11;7(11):e2442803. doi: 10.1001/jamanetworkopen.2024.42803 (PMC11555544; doi:10.1001/jamanetworkopen.2024.42803)
Supplement: Supplement 1. — eMethods. eTable 1. Longitudinal Sample (Baseline and 2-Year Follow-Up) Most Frequent Medications at Baseline eTable 2. Longitudinal Sample (Baseline and 2-Year Follow-Up) Medication Classes for Asthma Disease Management at Baseline eTable 3. Cross-Sectional Sample (2-year Follow-Up) Most Frequent Medications eTable 4. Cross-Sectional Sample (2-Year Follow-Up) Medication Classes for Asthma Disease Management eTable 5. Sample Derivation With Covariate Matching eTable 6. Longitudinal Sample Age and Pubertal Status at Baseline and 2-Year Follow-Up eTable 7. Longitudinal Analyses of Combined Parental Income Regressed on Race eTable 8. Longitudinal Analyses of Asthma Effects on Cognitive Outcomes Using a Matched Comparison Group eTable 9. Longitudinal Analyses of Asthma Effects on Cognitive Outcomes Using a Matched Comparison Group and Data-Driven Model Selection eTable 10. Longitudinal Sample Sensitivity Analyses Examining Developmental Trajectories of Cognitive Outcomes as a Function of Asthma Attacks eTable 11. Longitudinal Analyses of Asthma Effects on Cognitive Outcomes Across the ABCD Sample eTable 12. Cross-Sectional Sample Pubertal Status at 2-Year Follow-up eTable 13. Cross-Sectional Analyses of Combined Parental Income Regressed on Race eTable 14. Cross-Sectional Analyses of Asthma Effects on Cognitive Outcomes Using a Matched Comparison Group eTable 15. Cross-Sectional Analyses of Asthma Effects on Cognitive Outcomes Using a Matched Comparison Group and Data-Driven Model Selection eFigure 1. Longitudinal Sample (Baseline and 2-Year Follow-Up) Most Frequent Medications at Baseline eFigure 2. Cross-Sectional Sample (2-Year Follow-Up) Most Frequent Medications eFigure 3. Covariate Balance for Derivation of Asthma Comparison Groups eFigure 4. Longitudinal Sampling Procedure eFigure 5. Developmental trajectories of Processing Speed, and Inhibition and Attention eFigure 6. Longitudinal Sample Sensitivity Analyses Examining Developmental Trajectories of Cognitive Out [file jamanetwopen-e2442803-s001.pdf]

Supplemental Online Content

Christopher-Hayes NJ, Haynes SC, Kenyon NJ, Merchant VD, Schweitzer JB, Ghetti S. Asthma and memory function in children. *JAMA Netw Open*. 2024;7(11):e2442803. doi:10.1001/jamanetworkopen.2024.42803

eMethods.

eTable 1. Longitudinal Sample (Baseline and 2-Year Follow-Up) Most Frequent Medications at Baseline

eTable 2. Longitudinal Sample (Baseline and 2-Year Follow-Up) Medication Classes for Asthma Disease Management at Baseline

eTable 3. Cross-Sectional Sample (2-year Follow-Up) Most Frequent Medications

eTable 4. Cross-Sectional Sample (2-Year Follow-Up) Medication Classes for Asthma Disease Management

eTable 5. Sample Derivation With Covariate Matching

eTable 6. Longitudinal Sample Age and Pubertal Status at Baseline and 2-Year Follow-Up

eTable 7. Longitudinal Analyses of Combined Parental Income Regressed on Race

eTable 8. Longitudinal Analyses of Asthma Effects on Cognitive Outcomes Using a Matched Comparison Group

eTable 9. Longitudinal Analyses of Asthma Effects on Cognitive Outcomes Using a Matched Comparison Group and Data-Driven Model Selection

© 2024 Christopher-Hayes NJ et al. *JAMA Network Open.*

eTable 10. Longitudinal Sample Sensitivity Analyses Examining Developmental Trajectories of Cognitive Outcomes as a Function of Asthma Attacks

eTable 11. Longitudinal Analyses of Asthma Effects on Cognitive Outcomes Across the ABCD Sample

eTable 12. Cross-Sectional Sample Pubertal Status at 2-Year Follow-up

eTable 13. Cross-Sectional Analyses of Combined Parental Income Regressed on Race

eTable 14. Cross-Sectional Analyses of Asthma Effects on Cognitive Outcomes Using a Matched Comparison Group

eTable 15. Cross-Sectional Analyses of Asthma Effects on Cognitive Outcomes Using a Matched Comparison Group and Data-Driven Model Selection

eFigure 1. Longitudinal Sample (Baseline and 2-Year Follow-Up) Most Frequent Medications at Baseline

eFigure 2. Cross-Sectional Sample (2-Year Follow-Up) Most Frequent Medications

eFigure 3. Covariate Balance for Derivation of Asthma Comparison Groups

eFigure 4. Longitudinal Sampling Procedure

eFigure 5. Developmental trajectories of Processing Speed, and Inhibition and Attention

eFigure 6. Longitudinal Sample Sensitivity Analyses Examining Developmental Trajectories of Cognitive Outcomes as a Function of Asthma Attacks

eFigure 7. Cross-Sectional Sampling Procedure

eReferences.

This supplemental material has been provided by the authors to give readers additional information about their work.

© 2024 Christopher-Hayes NJ et al. *JAMA Network Open.*

eMethods 1

Our Supplemental Methods include information about sampling and matching procedures, and descriptive data concerning medication use across the longitudinal and cross-sectional samples. Below is the list of methods, tables, and figures by topic to orient the reader, but they are presented in this document by category (Methods, Tables, and Figures) in the order which they appear in the main manuscript.

Sampling and Matching Procedures
Method S1. Considerations for propensity score procedures.
Figure S3. Covariate balance for derivation of asthma comparison groups.
Table S5. Sample Derivation with Covariate Matching.
Figure S4. Flowchart of Longitudinal Sampling Procedure.
Figure S7. Flowchart of Cross-sectional Sampling Procedure.
Table S6. Longitudinal sample pubertal status at baseline and 2-year follow-up.
Table S7. Longitudinal Analyses of Combined Parental Income regressed on Race.
Table S12. Cross-sectional Sample Pubertal Status at 2-year Follow-up.
Table S13. Cross-sectional Analyses of Combined Parental Income regressed on Race.

Longitudinal and Cross-sectional Sample Medications
Table S1. Longitudinal sample (baseline & 2-year follow-up) most frequent medications at baseline.
Table S2. Longitudinal sample (baseline & 2-year follow-up) medication classes for asthma disease management at baseline.
Figure S1. Longitudinal sample (baseline & 2-year follow-up) most frequent medications at baseline.
Table S3. Cross-sectional sample (2-year follow-up) most frequent medications.
Table S4. Cross-sectional sample (2-year follow-up) medication classes for asthma disease management.
Figure S2. Cross-sectional sample (2-year follow-up) most frequent medications.

Results

Our Supplemental Results pertain to longitudinal and cross-section analyses. Below is the list of supplemental results by topic to orient the reader, but they are presented in this document by category (Tables, and Figures) in the order which they appear in the main manuscript.

Longitudinal Sample Analyses
Table S8. Longitudinal Analyses of Asthma Effects on Cognitive Outcomes using a Matched Comparison Group.
Figure S5. Developmental trajectories of Processing Speed, and Inhibition and Attention.
Table S9. Longitudinal Analyses of Asthma Effects on Cognitive Outcomes using a Matched Comparison Group and Data-driven Model Selection.
Table S10. Longitudinal Sample Sensitivity Analyses Examining Developmental Trajectories of Cognitive Outcomes as a Function of Asthma Attacks.
Figure S6. Longitudinal Sample Sensitivity Analyses Examining Developmental Trajectories of Cognitive Outcomes as a Function of Asthma Attacks.
Table S11. Longitudinal Analyses of Asthma Effects on Cognitive Outcomes Across the ABCD Sample.

Cross-sectional Sample Analyses
Table S14. Cross-sectional Analyses of Asthma Effects on Cognitive Outcomes using a Matched Comparison Group.
Table S15. Cross-sectional Analyses of Asthma Effects on Cognitive Outcomes using a Matched Comparison Group and Data-driven Model Selection.

© 2024 Christopher-Hayes NJ et al. *JAMA Network Open.*

eMethods 2

Method S1. Considerations for propensity score procedures.

The use of propensity score matching procedures attempt to minimize, but may not completely resolve, group differences in extraneous variables (Ho et al., 2011; Zhang et al., 2019), though study results using this matching approach of observational data have been shown to converges with those obtained by sampling of randomized trials (Anglemyer et al., 2014). Such procedures may also introduce bias by excluding participants (King and Nielsen, 2019). However, these analytical shortcomings are primarily an issue with smaller samples from which the comparison group is derived, where smaller samples may not be as representative of the general population, and where in smaller samples, adequate matching may be difficult to achieve. The present study used a large diverse sample that was well powered to perform matching procedures while minimizing bias, and most notably, the matching procedure yielded adequate balance for all extraneous covariates, for both longitudinal and cross-sectional analyses. The results using models that did not use matching procedures resulted in nearly identical longitudinal effects, indicating that the matching procedures implemented in the present study, if anything, improved estimation of effects of asthma on the cognitive outcomes of interest.

© 2024 Christopher-Hayes NJ et al. *JAMA Network Open.*

eTable 1. Longitudinal Sample (Baseline and 2-Year Follow-Up) Most Frequent Medications at Baseline

Earlier Onset (n = 135)			***Later Onset (n = 102)***			***Comparison (n = 237)***		
Medication	Count	Frequency	Medication	Count	Frequency	Medication	Count	Frequency
Albuterol	79	0.29	albuterol	24	0.12	zyrtec	17	0.04
Singulair	32	0.12	zyrtec	14	0.07	methylphenidate	17	0.04
Flovent	28	0.10	singulair	10	0.05	claritin	11	0.02
montelukast	25	0.09	vitamin	9	0.04	vyvanse	9	0.02
qvar	22	0.08	guanfacine	8	0.04	montelukast	9	0.02
zyrtec	19	0.07	flonase	7	0.03	flonase	8	0.02
proair	18	0.07	amoxicillin	7	0.03	adderall	8	0.02
ventolin	14	0.05	methylphenidate	7	0.03	vitamin	8	0.02
advair	14	0.05	montelukast	7	0.03	cetirizine	7	0.02
fluticasone	14	0.05	melatonin	6	0.03	lexapro	7	0.02
benadryl	11	0.04	qvar	6	0.03	melatonin	6	0.01
flonase	10	0.04	benadryl	5	0.02	ibuprofen	6	0.01
claritin	10	0.04	concerta	5	0.02	benadryl	5	0.01
symbicort	9	0.03	claritin	5	0.02	amoxicillin	4	0.01
vitamin	8	0.03	vyvanse	5	0.02	singulair	4	0.01

© 2024 Christopher-Hayes NJ et al. *JAMA Network Open.*

eTable 2. Longitudinal Sample (Baseline and 2-Year Follow-Up) Medication Classes for Asthma Disease Management at Baseline

	Earlier Onset (n = 135)		***Later Onset (n = 102)***		***Comparison (n = 237)***		***χ2***	***df***	***p-value***
Medication Group	Count	Frequency	Count	Frequency	Count	Frequency			
Bronchodialator	60	0.44	5	0.05	3	0.01	139.93	2	0.000***
Corticosteroids	38	0.28	6	0.06	11	0.05	50.482	2	0.000***
Antiinflamm	32	0.24	6	0.06	7	0.03	45.072	2	0.000***

Bronchodialator: albuterol, proair, ventolin, levalbuterol, formoterol, salmeterol, tiotropium, proventil
Corticosteroids: fluticasone, flunisolide, budesonide, pulmicort, beclometasone, beclomethasone, qvar, ciclesonide, mometasone, triamcinolone, flovent, flonase, prednisone
Antiinflammatory: montelukast, singulair
All statistical tests used Pearson's Chi-squared test. All results were such that: Earlier A > Later A > Comparison.
*p < 0.05; **p < 0.01; ***p < 0.001

© 2024 Christopher-Hayes NJ et al. *JAMA Network Open.*

eTable 3. Cross-Sectional Sample (2-year Follow-Up) Most Frequent Medications

Asthma (n = 1031)			*Comparison (n = 1031)*		
Medication	Count	Frequency	Medication	Count	Frequency
albuterol	146	0.14	zyrtec	31	0.03
Zyrtec	66	0.06	methylphenidate	24	0.02
singulair	56	0.05	adderall	22	0.02
Flovent	44	0.04	concerta	20	0.02
montelukast	44	0.04	vyvanse	17	0.02
Qvar	40	0.04	montelukast	14	0.01
Flonase	28	0.03	flonase	12	0.01
concerta	23	0.02	cetirizine	12	0.01
melatonin	20	0.02	claritin	11	0.01
symbicort	20	0.02	ibuprofen	10	0.01
adderall	20	0.02	vitamin	10	0.01
Claritin	18	0.02	tylenol	9	0.01
cetirizine	17	0.02	clonidine	9	0.01
Advair	17	0.02	amoxicillin	9	0.01
fluticasone	17	0.02	singulair	8	0.01

© 2024 Christopher-Hayes NJ et al. *JAMA Network Open.*

eTable 4. Cross-Sectional Sample (2-Year Follow-Up) Medication Classes for Asthma Disease Management

	Asthma (n = 1031)		***Comparison (n = 1031)***				
Medication Group	Count	Frequency	Count	Frequency	***χ2***	***df***	***p-value***
Bronchodialator	167	0.16	3	0.00	170.33	1	0.000***
Corticosteroids	121	0.12	22	0.02	76.128	1	0.000***
Antiinflamm	88	0.09	19	0.02	45.58	1	0.000***

Bronchodialator: albuterol, proair, ventolin, levalbuterol, formoterol, salmeterol, tiotropium, proventil
Corticosteroids: fluticasone, flunisolide, budesonide, pulmicort, beclometasone, beclomethasone, qvar, ciclesonide, mometasone, triamcinolone, flovent, flonase, prednisone
Antiinflammatory: montelukast, singulair
All statistical tests used Pearson's Chi-squared test. All results were such that: Asthma > Comparison.
*p < 0.05; **p < 0.01; ***p < 0.001

© 2024 Christopher-Hayes NJ et al. *JAMA Network Open.*

eTable 5. Sample Derivation With Covariate Matching

Longitudinal covariate matching at baseline

	n = Non-Asthma	n = Asthma
All	8414	237
Matched	237	237
Unmatched	8177	0

Cross-sectional covariate matching at 2-year follow-up

	n = Non-Asthma	n = Asthma
All	7344	1031
Matched	1031	1031
Unmatched	6313	0

© 2024 Christopher-Hayes NJ et al. *JAMA Network Open.*

eTable 6. Longitudinal Sample Age and Pubertal Status at Baseline and 2-Year Follow-Up

Pubertal Metric	*F*	*df*	*p-value*
Baseline			
Age	0.04	2	0.961
Salivary DHEA	0.12	2	0.888
Combined Parental and Youth Peterson Pubertal Development Scale	1.25	2	0.289
2-year			
Age	0.38	2	0.681
Salivary DHEA	1.48	2	0.229
Combined Parental and Youth Peterson Pubertal Development Scale	1.17	2	0.313

*p < 0.05

© 2024 Christopher-Hayes NJ et al. *JAMA Network Open.*

eTable 7. Longitudinal Analyses of Combined Parental Income Regressed on Race

Baseline:
n = 472

term	*df*	*β*	*2.5%*	*97.5%*	*p-value*
(Intercept)	468	5.8232	5.227	6.4193	0.000***
American Indian	468	-0.4553	-1.1404	0.2297	0.192
Asian	468	0.3295	-0.3602	1.0191	0.348
Black	468	-0.7691	-1.3319	-0.2062	0.008**
Other Race	468	0.8275	-0.7764	2.4314	0.311
White	468	1.8219	1.2343	2.4096	0.000***

*p < 0.05; **p < 0.01; ***p < 0.001

© 2024 Christopher-Hayes NJ et al. *JAMA Network Open.*

eTable 8. Longitudinal Analyses of Asthma Effects on Cognitive Outcomes Using a Matched Comparison Group

Baseline: n = 472	***term***	***df***	***df(resid)***	***β***	***2.5%***	***97.5%***	***p-value***
EPISODIC MEMORY	(Intercept)	590.5616	898	-0.7758	-1.0683	-0.4832	0.000***
	Age (T0)	463.4574	898	0.1244	0.0061	0.2427	0.039*
	ΔAge	462.4531	898	0.2798	0.2101	0.3496	0.000***
	Sex (female)	461.5224	898	-0.2152	-0.3574	-0.073	0.003**
	Income	595.7911	898	0.0728	0.0433	0.1022	0.000***
	Other Health	464.0538	898	0.0288	-0.1277	0.1853	0.718
	Later Onset	805.7133	898	-0.1211	-0.3414	0.0992	0.281
	Earlier Onset	811.2719	898	0.0934	-0.1099	0.2966	0.367
	Later x ΔAge	453.8916	898	-0.0204	-0.1454	0.1046	0.749
	Earlier x ΔAge	454.7831	898	-0.1683	-0.2849	-0.0517	0.005**
PROCESSING SPEED	(Intercept)	583.1691	848	-0.8786	-1.1591	-0.598	0.000***
	Age (T0)	461.0237	848	0.3371	0.2236	0.4507	0.000***
	ΔAge	447.1766	848	0.385	0.3158	0.4542	0.000***
	Sex (female)	461.5916	848	-0.0884	-0.2249	0.0482	0.204
	Income	598.3137	848	0.025	-0.0032	0.0531	0.0824
	Other Health	474.3326	848	0.0883	-0.0631	0.2397	0.252
	Later Onset	754.7232	848	-0.0272	-0.2328	0.1785	0.795
	Earlier Onset	758.7748	848	-0.0685	-0.258	0.1211	0.478
	Later x ΔAge	434.515	848	0.0248	-0.0986	0.1482	0.693
	Earlier x ΔAge	425.8697	848	0.0716	-0.0387	0.182	0.203
INHIBITION & ATTENTION	(Intercept)	578.721	847	-0.8701	-1.1637	-0.5764	0.000***
	Age (T0)	456.3077	847	0.3072	0.1885	0.4258	0.000***
	ΔAge	443.421	847	0.2875	0.2141	0.3609	0.000***
	Sex (female)	456.9982	847	-0.0556	-0.1983	0.0872	0.445
	Income	590.7614	847	0.0407	0.0112	0.0702	0.007**
	Other Health	469.8572	847	0.0865	-0.0717	0.2448	0.283
	Later Onset	758.5483	847	0.0845	-0.1314	0.3003	0.443
	Earlier Onset	762.3916	847	-0.1818	-0.3808	0.0171	0.073
	Later x ΔAge	430.5473	847	-0.0213	-0.1521	0.1096	0.749
	Earlier x ΔAge	422.5904	847	0.0768	-0.0406	0.1942	0.199

Random effects: (1 | subID)
*p < 0.05; **p < 0.01; ***p < 0.001

© 2024 Christopher-Hayes NJ et al. *JAMA Network Open.*

eTable 9. Longitudinal Analyses of Asthma Effects on Cognitive Outcomes Using a Matched Comparison Group and Data-Driven Model Selection

Baseline: n = 472	*term*	*df*	*df(resid)*	*β*	*2.5%*	*97.5%*	*p-value*
EPISODIC MEMORY	(Intercept)	603.3959	899	-0.757	-1.031	-0.483	0.000***
	Age (T0)	463.793	899	0.1278	0.0111	0.2446	0.032*
	ΔAge	462.5957	899	0.2798	0.2101	0.3496	0.000***
	Sex (female)	462.7385	899	-0.214	-0.3559	-0.0721	0.003**
	Income	595.7622	899	0.0724	0.0431	0.1017	0.000***
	Other Health						
	Later Onset	807.7109	899	-0.1219	-0.3419	0.0982	0.278
	Earlier Onset	812.9499	899	0.0942	-0.1088	0.2973	0.363
	Later x ΔAge	454.0808	899	-0.0206	-0.1456	0.1043	0.746
	Earlier x ΔAge	455.0185	899	-0.1686	-0.2852	-0.052	0.005**
PROCESSING SPEED	(Intercept)	562.9242	855	-0.718	-0.8537	-0.5824	0.000***
	Age (T0)	464.3461	855	0.3437	0.2316	0.4558	0.000***
	ΔAge	434.9494	855	0.4139	0.3664	0.4615	0.000***
	Sex (female)						
	Income						
	Other Health						
	Later Onset						
	Earlier Onset						
	Later x ΔAge						
	Earlier x ΔAge						
INHIBITION & ATTENTION	(Intercept)	574.6199	853	-0.9015	-1.1491	-0.6539	0.000***
	Age (T0)	458.1933	853	0.3134	0.1964	0.4304	0.000***
	ΔAge	432.0207	853	0.3048	0.2541	0.3555	0.000***
	Sex (female)						
	Income	588.7654	853	0.0441	0.0152	0.0731	0.003**
	Other Health						
	Later Onset						
	Earlier Onset						
	Later x ΔAge						
	Earlier x ΔAge						

Cells shaded gray represent covariates eliminated by data-driven model selection.
Random effects: (1 | subID)
*p < 0.05; **p < 0.01; ***p < 0.001

© 2024 Christopher-Hayes NJ et al. *JAMA Network Open.*

eTable 10. Longitudinal Sample Sensitivity Analyses Examining Developmental Trajectories of Cognitive Outcomes as a Function of Asthma Attacks

Baseline: n = 472	*term*	*df*	*df(resid)*	*β*	*2.5%*	*97.5%*	*p-value*
EPISODIC MEMORY	(Intercept)	572.9849	831	-0.8198	-1.105	-0.5347	0.000***
	Age (T0)	466.8416	831	0.1411	0.0206	0.2616	0.022
	ΔAge	495.7522	831	0.2757	0.2128	0.3386	0.000***
	Sex (female)	466.5788	831	-0.1885	-0.3332	-0.0437	0.011
	Income	583.8223	831	0.0744	0.0449	0.1039	0.000***
	Other Health	477.0076	831	-0.0011	-0.1616	0.1595	0.990
	Attacks	759.2874	831	0.0211	-0.0074	0.0496	0.146
	Attacks x ΔAge	659.8683	831	-0.0589	-0.106	-0.0118	0.014*
PROCESSING SPEED	(Intercept)	563.4695	804	-0.9009	-1.1724	-0.6294	0.000***
	Age (T0)	457.8318	804	0.3474	0.2324	0.4624	0.000***
	ΔAge	464.0844	804	0.4078	0.3475	0.468	0.000***
	Sex (female)	460.6409	804	-0.0772	-0.2154	0.0611	0.273
	Income	580.4977	804	0.023	-0.0051	0.0511	0.108
	Other Health	478.8541	804	0.0954	-0.0586	0.2493	0.224
	Attacks	724.3552	804	-0.0099	-0.0363	0.0166	0.465
	Attacks x ΔAge	609.0213	804	0.0138	-0.0303	0.0578	0.540
INHIBITION & ATTENTION	(Intercept)	554.3349	803	-0.8945	-1.1798	-0.6092	0.000***
	Age (T0)	448.7512	803	0.3209	0.2004	0.4415	0.000***
	ΔAge	459.6468	803	0.297	0.2325	0.3614	0.000***
	Sex (female)	451.8705	803	-0.0627	-0.2077	0.0822	0.395
	Income	568.168	803	0.041	0.0115	0.0705	0.007**
	Other Health	470.3689	803	0.1104	-0.051	0.2719	0.179
	Attacks	728.6557	803	-0.0355	-0.0636	-0.0074	0.014*
	Attacks x ΔAge	611.7992	803	0.0105	-0.0364	0.0575	0.659

Random effects: (1 | subID)
*p < 0.05; **p < 0.01; ***p < 0.001

© 2024 Christopher-Hayes NJ et al. *JAMA Network Open.*

eTable 11. Longitudinal Analyses of Asthma Effects on Cognitive Outcomes Across the ABCD Sample

Baseline: n = 472	*term*	*df*	*df(resid)*	*β*	*2.5%*	*97.5%*	*p-value*
EPISODIC MEMORY	(Intercept)	13098.3992	19196	-0.8656	-0.9201	-0.8111	0.000***
	Age (T0)	10377.3685	19196	0.1481	0.1232	0.173	0.000***
	ΔAge	9744.2227	19196	0.2037	0.1935	0.214	0.000***
	Sex (female)	10368.5039	19196	-0.1173	-0.1484	-0.0863	0.000***
	Income	13833.939	19196	0.0805	0.0745	0.0865	0.000***
	Other Health	10122.0602	19196	-0.0189	-0.062	0.0242	0.391
	Later Onset	16572.3017	19196	-0.031	-0.1928	0.1308	0.707
	Earlier Onset	16736.7787	19196	0.1298	-0.0171	0.2767	0.083
	Later x ΔAge	9121.0013	19196	0.0167	-0.069	0.1024	0.702
	Earlier x ΔAge	9145.1874	19196	-0.1248	-0.2035	-0.0461	0.002**
PROCESSING SPEED	(Intercept)	12581.8119	17446	-0.8898	-0.9413	-0.8382	0.000***
	Age (T0)	10263.9318	17446	0.3036	0.2799	0.3272	0.000***
	ΔAge	8517.2752	17446	0.4466	0.4364	0.4569	0.000***
	Sex (female)	10216.0738	17446	-0.1252	-0.1547	-0.0957	0.000***
	Income	13385.6294	17446	0.0389	0.0331	0.0446	0.000***
	Other Health	9870.0759	17446	0.0103	-0.0305	0.0511	0.622
	Later Onset	14986.9585	17446	-0.0292	-0.1772	0.1187	0.699
	Earlier Onset	15129.9951	17446	-0.0114	-0.1456	0.1228	0.867
	Later x ΔAge	7712.2248	17446	-0.016	-0.0977	0.0658	0.702
	Earlier x ΔAge	7489.5443	17446	-0.0085	-0.0793	0.0624	0.815
INHIBITION & ATTENTION	(Intercept)	12206.9656	17496	-1.0238	-1.0785	-0.9692	0.000***
	Age (T0)	9847.9564	17496	0.2148	0.1898	0.2398	0.000***
	ΔAge	8210.3717	17496	0.3138	0.3027	0.3249	0.000***
	Sex (female)	9801.4015	17496	0.0369	0.0058	0.0681	0.020*
	Income	12965.1887	17496	0.0716	0.0656	0.0777	0.000***
	Other Health	9431.6317	17496	0.0411	-0.002	0.0842	0.062
	Later Onset	15057.0156	17496	0.16	0.0024	0.3176	0.047*
	Earlier Onset	15196.6307	17496	-0.1088	-0.2518	0.0342	0.136
	Later x ΔAge	7394.4159	17496	-0.0468	-0.1358	0.0422	0.303
	Earlier x ΔAge	7184.7712	17496	0.0264	-0.0511	0.1038	0.504

*p < 0.05; **p < 0.01; ***p < 0.001

© 2024 Christopher-Hayes NJ et al. *JAMA Network Open.*

eTable 12. Cross-Sectional Sample Pubertal Status at 2-Year Follow-up

Pubertal Metric	***F***	***df***	***p-value***
2-year			
Salivary DHEA	0.08	1	0.779
Combined Parental and Youth Peterson Pubertal Development Scale	0.06	1	0.815

*p < 0.05

© 2024 Christopher-Hayes NJ et al. *JAMA Network Open.*

eTable 13. Cross-Sectional Analyses of Combined Parental Income Regressed on Race

Baseline:
n = 2,062

term	*df*	*β*	*2.5%*	*97.5%*	*p-value*
(Intercept)	2056	5.8761	5.5605	6.1918	0.000***
American Indian	2056	-0.3921	-0.8202	0.036	0.073
Asian	2056	0.7137	0.3002	1.1272	0.000***
Black	2056	-1.0569	-1.3561	-0.7576	0.000***
Other Race	2056	0.261	-0.7135	1.2355	0.5995
White	2056	1.5421	1.233	1.8511	0.000***

*p < 0.05; **p < 0.01; ***p < 0.001

© 2024 Christopher-Hayes NJ et al. *JAMA Network Open.*

eTable 14. Cross-Sectional Analyses of Asthma Effects on Cognitive Outcomes Using a Matched Comparison Group

2-Year Follow-up: n = 2,062	*term*	*df*	*df(resid)*	*β*	*2.5%*	*97.5%*	*p-value*
EPISODIC MEMORY	(Intercept)	1609.97	1946	-1.0549	-1.8719	-0.238	0.011*
	Age	1781.8807	1946	0.0046	-0.001	0.0102	0.109
	Sex (female)	1885.4171	1946	-0.0988	-0.1864	-0.0112	0.027*
	Income	1168.5144	1946	0.0739	0.0564	0.0914	0.000***
	Other Health	1934.2067	1946	-0.0048	-0.0922	0.0826	0.914
	Asthma	1936.7586	1946	-0.0933	-0.1804	-0.0061	0.036*
PROCESSING SPEED	(Intercept)	1457.7829	1575	-3.3843	-4.3035	-2.4652	0.000***
	Age	1527.7509	1575	0.0231	0.0168	0.0295	0.000***
	Sex (female)	1520.6615	1575	-0.1648	-0.2615	-0.0681	0.000***
	Income	1222.5616	1575	0.034	0.0139	0.054	0.000***
	Other Health	1568.7268	1575	-0.0063	-0.1032	0.0907	0.8992
	Asthma	1569.0142	1575	-0.1268	-0.2228	-0.0309	0.009**
INHIBITION & ATTENTION	(Intercept)	1455.3467	1585	-1.5275	-2.4476	-0.6074	0.001**
	Age	1537.1308	1585	0.0073	9e-04	0.0136	0.025*
	Sex (female)	1536.8646	1585	0.073	-0.0239	0.1698	0.1397
	Income	1181.2449	1585	0.068	0.0479	0.088	0.000***
	Other Health	1581.5985	1585	0.0748	-0.0221	0.1718	0.130
	Asthma	1581.3282	1585	-0.1143	-0.2103	-0.0183	0.020*

Random effects: (1 | site_id_l/rel_family_id)
*p < 0.05; **p < 0.01; ***p < 0.001

© 2024 Christopher-Hayes NJ et al. *JAMA Network Open.*

eTable 15. Cross-Sectional Analyses of Asthma Effects on Cognitive Outcomes Using a Matched Comparison Group and Data-Driven Model Selection

2-Year Follow-up: n = 2,062	*term*	*df*	*df(resid)*	*β*	*2.5%*	*97.5%*	*p-value*
EPISODIC MEMORY	(Intercept)	365.2567	1949	-0.4046	-0.5494	-0.2597	0.000***
	Age						
	Sex (female)	1948.5125	1949	-0.0951	-0.1825	-0.0078	0.033*
	Income	1171.5105	1949	0.0748	0.0574	0.0922	0.000***
	Other Health						
	Asthma	1950.7031	1949	-0.0951	-0.1821	-0.0081	0.032*
PROCESSING SPEED	(Intercept)	1499.345	1577	-3.3811	-4.2945	-2.4676	0.000***
	Age	1564.9237	1577	0.0231	0.0168	0.0294	0.000***
	Sex (female)	1570.5809	1577	-0.1647	-0.2609	-0.0684	0.000***
	Income	1293.0909	1577	0.034	0.0141	0.054	0.000***
	Other Health						
	Asthma	1574.0615	1577	-0.1247	-0.2205	-0.0289	0.011*
INHIBITION & ATTENTION	(Intercept)	1457.4616	1587	-1.4894	-2.4086	-0.5703	0.002**
	Age	1537.5176	1587	0.0076	0.0012	0.0139	0.019*
	Sex (female)						
	Income	1181.3821	1587	0.0683	0.0483	0.0884	0.000***
	Other Health						
	Asthma	1583.209	1587	-0.1109	-0.2069	-0.0149	0.024*

Cells shaded gray represent covariates eliminated by data-driven model selection.
Random effects: (1 | site_id_l/rel_family_id).
site_id_l was retained for all three models. rel_family_id was retained only for Inhibition and Attention.
*p < 0.05; **p < 0.01; ***p < 0.001

© 2024 Christopher-Hayes NJ et al. *JAMA Network Open.*

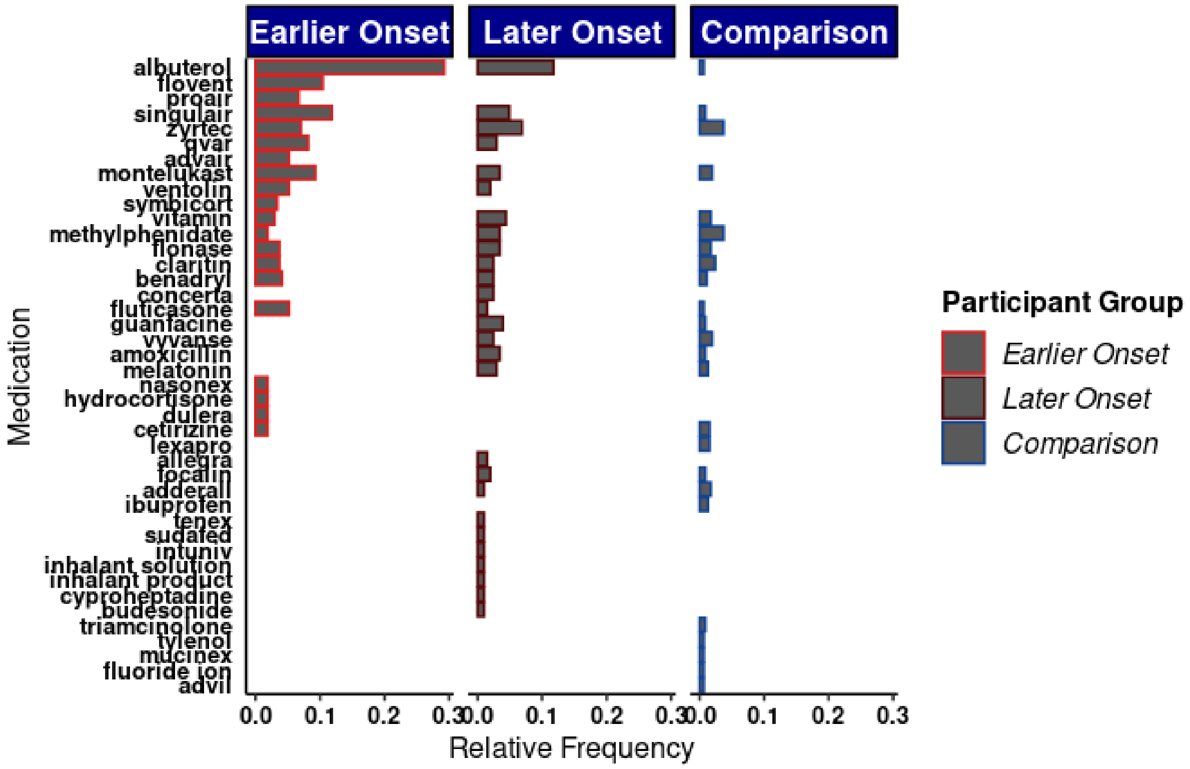

eFigure 1. Longitudinal Sample (Baseline and 2-Year Follow-Up) Most Frequent Medications at Baseline

© 2024 Christopher-Hayes NJ et al. *JAMA Network Open.*

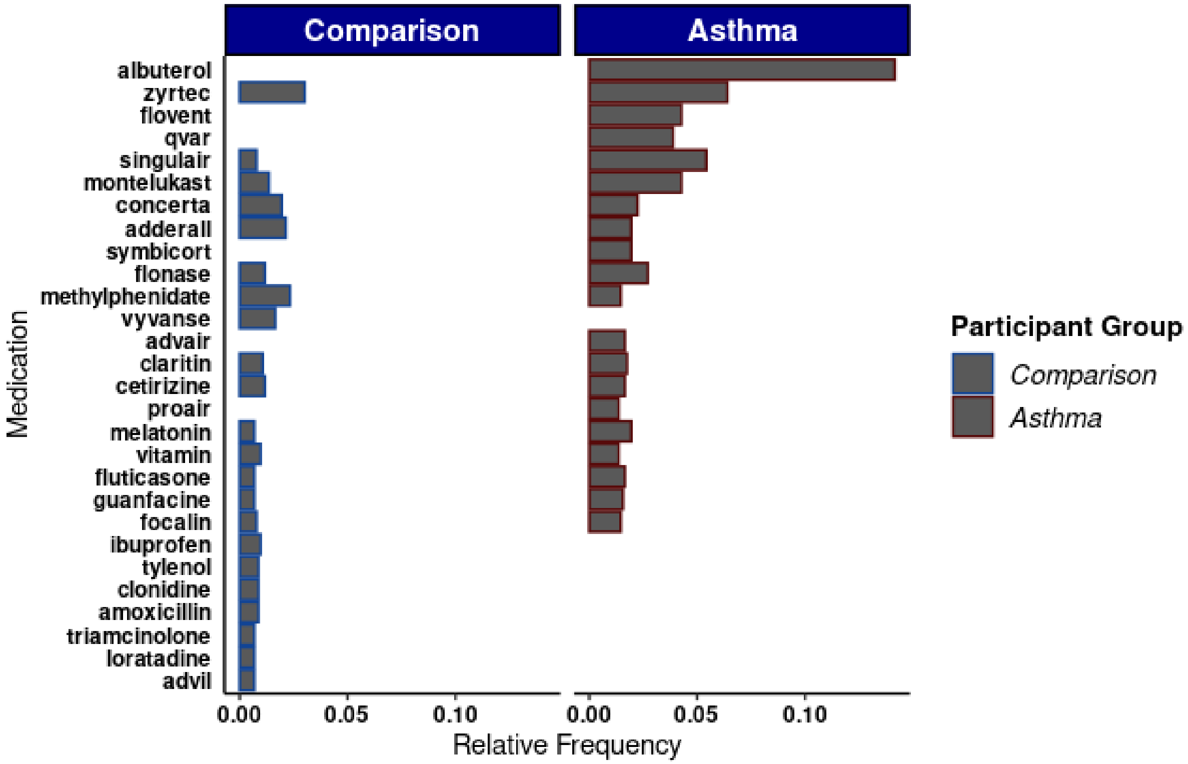

eFigure 2. Cross-Sectional Sample (2-Year Follow-Up) Most Frequent Medications

© 2024 Christopher-Hayes NJ et al. *JAMA Network Open.*

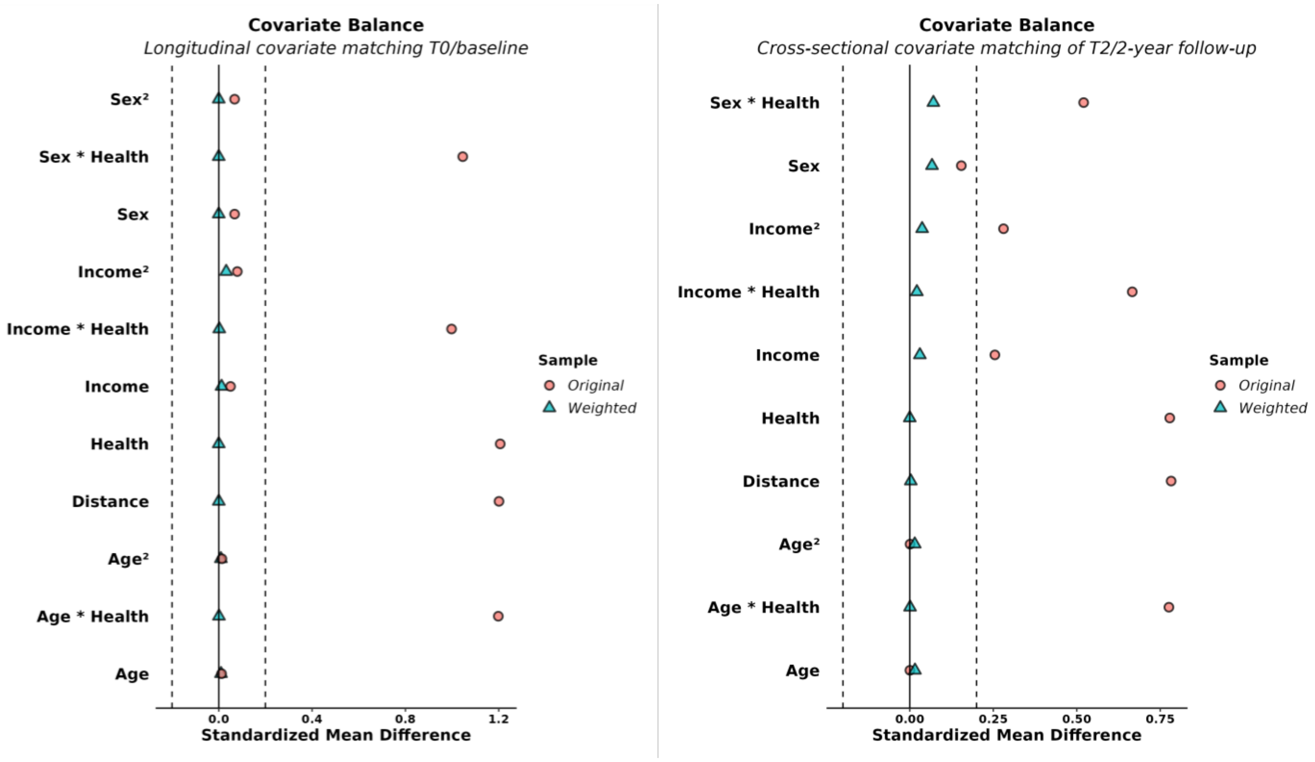

eFigure 3. Covariate Balance for Derivation of Asthma Comparison Groups Love plots graphically displaying covariate balance before and after propensity score matching for both *Left)* cross-sectional and *Right)* longitudinal samples. Colors and shapes are doubly indicative of the status of the study sample between stages of matching procedure (Circles, Salmon = Pre-match Original Sample, Triangles, Turquoise = Post-match Weighted Sample). Covariates included in the matching procedures are denoted on the y-axis, and standardized mean differences per each covariate term are shown on the x-axis. All main effects of covariate terms are displayed. Interaction terms of covariates are displayed if the standardized mean difference of the original sample exceeded the balance threshold. The standardized mean difference threshold (|0.2|) is marked with vertically dashed lines; points that fall within the dashed lines are adequately balanced, whereas points outside of the dashed lines are unbalanced.

© 2024 Christopher-Hayes NJ et al. *JAMA Network Open.*

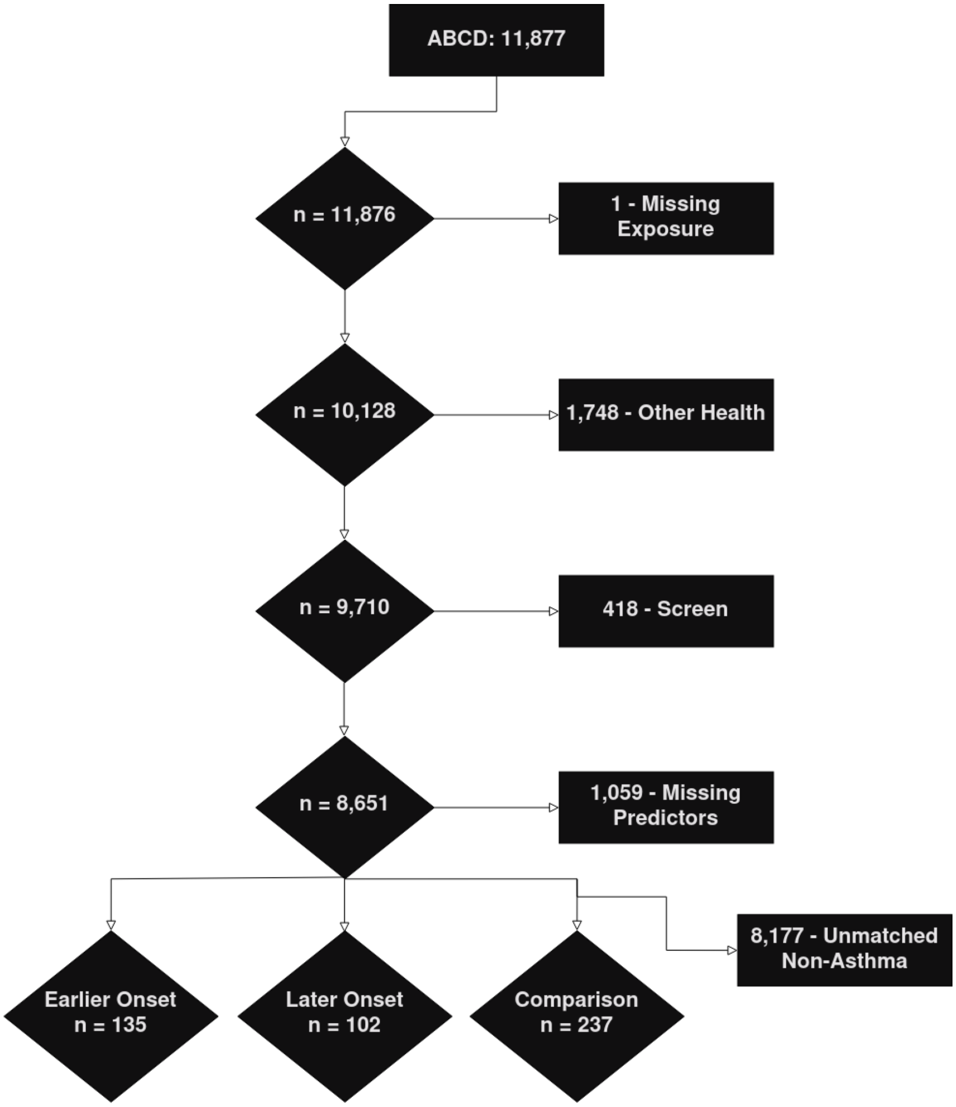

eFigure 4. Longitudinal Sampling Procedure Sankey diagram to visualize the sampling procedure used for longitudinal analyses. “ABCD” = the ABCD Study population at baseline; “Missing Exposure” = missing data for asthma indicators; “Other Health” = a positive indicator for exclusionary health conditions; “Screen” = a positive indicator for other exclusionary conditions; “Missing Predictors” = missing data of covariates used for matching procedures.

© 2024 Christopher-Hayes NJ et al. *JAMA Network Open.*

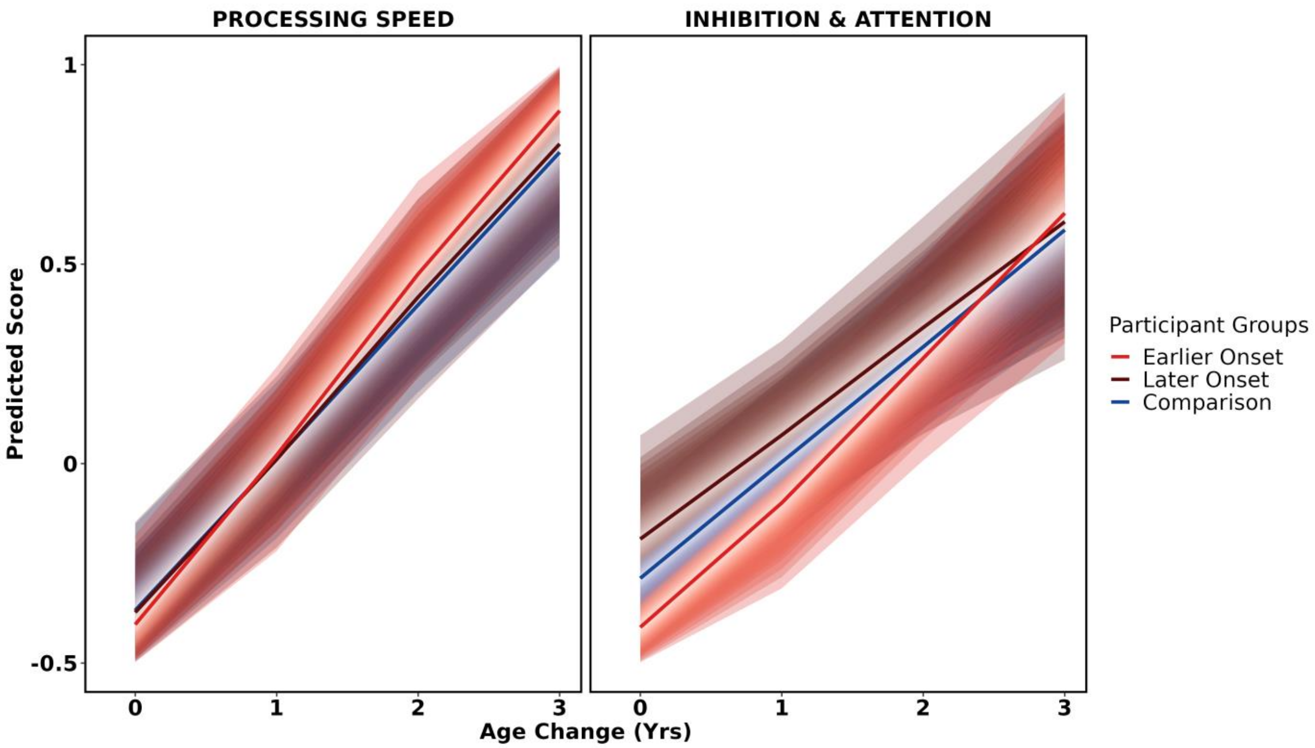

eFigure 5. Developmental trajectories of Processing Speed, and Inhibition and Attention Interactions between participant group and time with lines-of-best-fit for fixed effects and a gradient underlay of bootstrapped interval estimates (nsim = 1000) around prediction lines (Light Red = Earlier Onset Asthma, Maroon = Later Onset Asthma, Blue = Comparison). Change in age (years) from the baseline timepoint is denoted on the x-axis, and scaled predicted scores for each respective cognitive measure are shown on the y-axis.

© 2024 Christopher-Hayes NJ et al. *JAMA Network Open.*

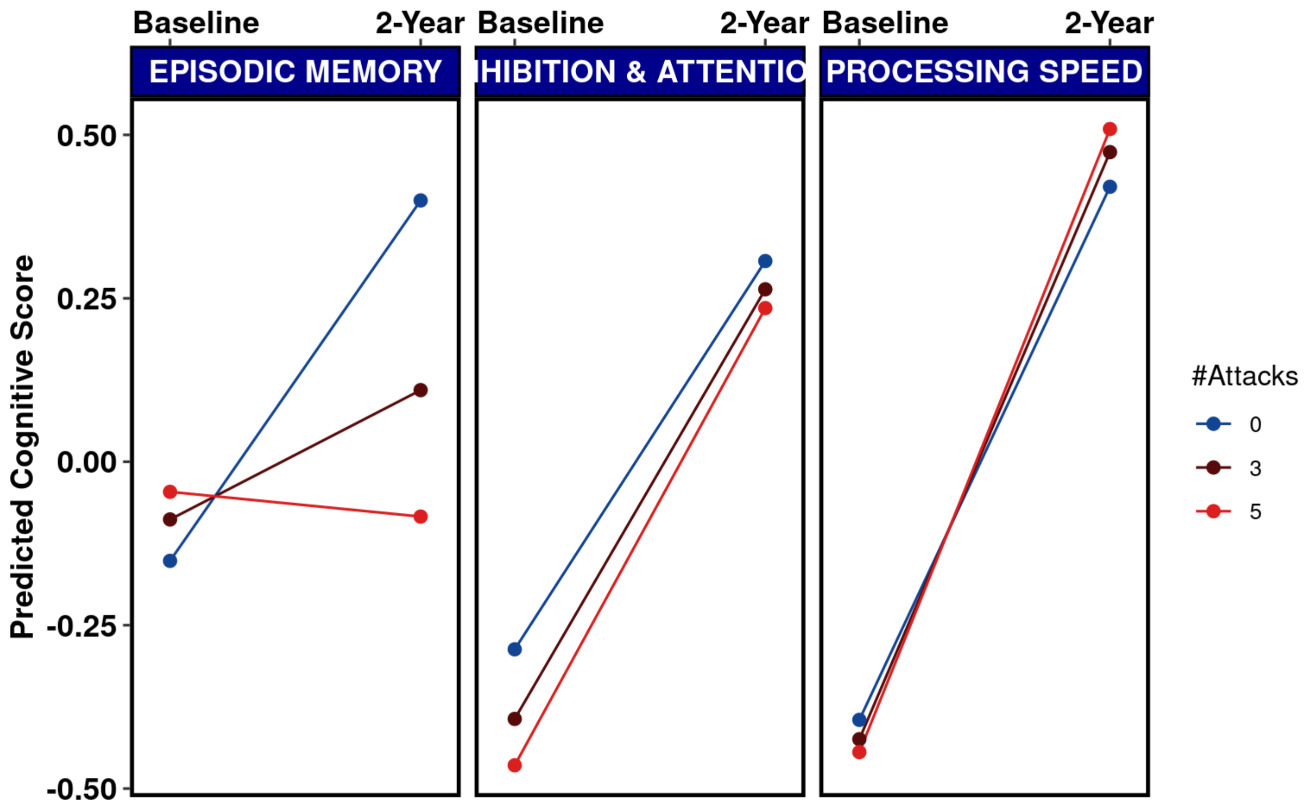

eFigure 6. Longitudinal Sample Sensitivity Analyses Examining Developmental Trajectories of Cognitive Outcomes as a Function of Asthma Attacks We found a significant interaction between change in age and number of asthma attacks on episodic memory but not processing speed or inhibition and attention, suggesting that more severe asthma is associated with more reduced rates of improvement in memory performance. Interaction between number of asthma attacks (0, 1, 4) and time with lines-of-best-fit for fixed effects and 95% confidence interval around prediction lines (Light Red = Earlier Onset Asthma, Maroon = Later Onset Asthma, Blue = Comparison). Change in age (years) from the baseline timepoint is denoted on the x-axis, and scaled predicted scores for episodic memory are shown on the y-axis.

© 2024 Christopher-Hayes NJ et al. *JAMA Network Open.*

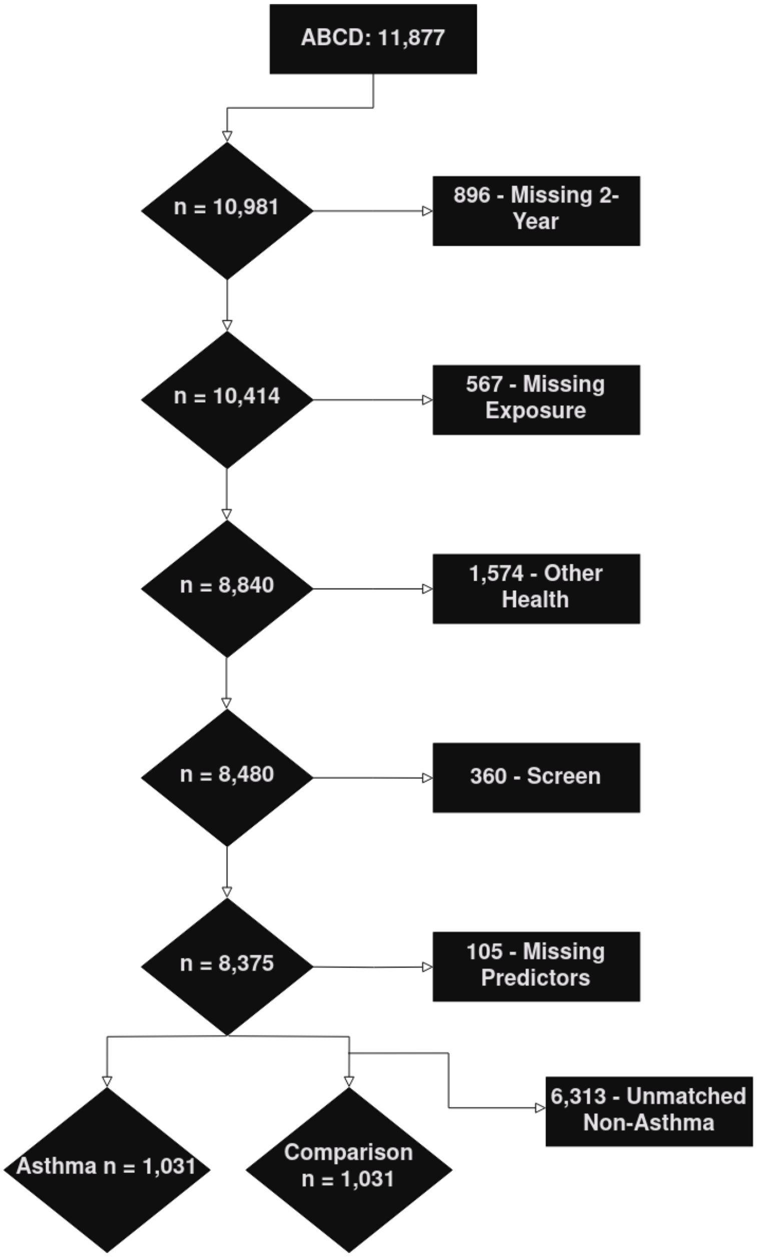

eFigure 7. Cross-Sectional Sampling Procedure Sankey diagram to visualize the sampling procedure used for cross-sectional analyses. “ABCD” = the ABCD Study population at baseline; “2-Year-Follow-Up” = the ABCD Study population at the 2-year-follow-up timepoint; “Missing Exposure” = missing data for asthma indicators; “Other Health” = a positive indicator for exclusionary health conditions; “Screen” = a positive indicator for other exclusionary conditions; “Missing Predictors” = missing data of covariates used for matching procedures.

© 2024 Christopher-Hayes NJ et al. *JAMA Network Open.*

eReferences

Anglemyer, A., Horvath, H.T., Bero, L., 2014. Healthcare outcomes assessed with observational study designs compared with those assessed in randomized trials. Cochrane Database of Systematic Reviews. https://doi.org/10.1002/14651858.MR000034.pub2

Ho, D., Imai, K., King, G., Stuart, E.A., 2011. MatchIt: Nonparametric Preprocessing for Parametric Causal Inference. Journal of Statistical Software 42, 1–28. https://doi.org/10.18637/jss.v042.i08

King, G., Nielsen, R., 2019. Why Propensity Scores Should Not Be Used for Matching. Political Analysis 27, 435–454.

Zhang, Z., Kim, H.J., Lonjon, G., Zhu, Y., 2019. Balance diagnostics after propensity score matching. Ann Transl Med 7, 16. https://doi.org/10.21037/atm.2018.12.10

© 2024 Christopher-Hayes NJ et al. *JAMA Network Open.*
