# Supplementary material for: Asthma and Memory Function in Children
Source: JAMA Netw Open. 2024 Nov 11;7(11):e2442803. doi: 10.1001/jamanetworkopen.2024.42803 (PMC11555544; doi:10.1001/jamanetworkopen.2024.42803)
Supplement: Supplement 2. — Data Sharing Statement [file jamanetwopen-e2442803-s002.pdf]

Data Sharing Statement

Christopher-Hayes. Asthma and Memory Function in Children. *JAMA Netw Open*. Published November 05, 2024. doi:10.1001/jamanetworkopen.2024.42803

Data
Data available: Yes
Data types: Other (please specify)
Additional Information: Data used in the preparation of this article were obtained from the Adolescent Brain Cognitive DevelopmentSM (ABCD) Study (https://abcdstudy.org), held in the NIMH Data Archive (NDA). The ABCD Study® data repository grows and changes over time.
How to access data: https://abcdstudy.org
When available: With publication

Supporting Documents
Document types: Statistical/analytic code
How to access documents: nichrishayes@gmail.com
When available: With publication

Additional Information
Who can access the data: Researchers whose proposed use of the data has been approved.
Types of analyses: For any purpose.
Mechanisms of data availability: With investigator support.
